# Kidney Autotransplantation for Treatment of Ureteric Obstruction: A Case Report and Brief Review of the Literature

**DOI:** 10.1155/2021/6646958

**Published:** 2021-07-21

**Authors:** Parth Joshi, Joanne Lin, Tej Sura, Posan S. Limbu, Vatche Melkonian, Bahar Bastani, Chintalapati Varma

**Affiliations:** Saint Louis University, School of Medicine, Saint Louis, Missouri, USA

## Abstract

Autologous kidney transplantation is a relatively rare procedure that has been used as an alternative treatment for a variety of complex genitourinary problems, in particular for the treatment of complex proximal ureteral strictures. In this case report, a 47-year-old male, who had undergone a living donor nephrectomy 14 years earlier, presented with episodes of acute kidney injury on chronic kidney disease. He was found to have a complex proximal ureter stricture of his solitary right kidney. He underwent nephrectomy with subsequent autotransplantation of the kidney into the right iliac fossa. His renal function improved significantly after surgery. Renal autotransplantation may be considered for the management of proximal ureteral obstruction when alternative options are contraindicated.

## 1. Introduction

The first reported case of kidney autologous transplantation (KAT) in humans was in 1961 in a patient who was suffering from unilateral renal artery stenosis [1, 2]. Since that time, it has been used as an alternate treatment for a variety of complex renal issues. The most common indication for KAT has been for the treatment of complex and proximal ureteral strictures [3, 4]. KAT itself is a relatively rare procedure. One study using a national inpatient sample database found that there had only been 817 patients who had undergone KAT between 2002 and 2012 [5].

Obstructive uropathy refers to a condition in which the presence of abnormal structural anatomy or altered function affects the kidney's baseline ability to excrete urine. There are multiple etiologies of obstructive uropathy, and it is possible for obstruction to occur anywhere along the urinary tract. The obstruction can be classified as extrinsic, intrinsic, or “functional” obstruction due to an aperistaltic segment of the ureter. Intrinsic causes can include calculous disease, ureteral neoplasms, and strictures. Extrinsic causes include obstruction of the ureteropelvic junction due to a crossing vessel, retroperitoneal fibrosis, and retroperitoneal malignancy [6].

Obstructive uropathy can progress into chronic kidney disease (CKD) and potentially end-stage renal disease (ESRD) when it occurs either bilaterally or in patients with only one functioning kidney at baseline if it is not recognized early and treated appropriately.

In this case report, we describe an autologous kidney transplant performed on a patient with obstructive uropathy in the unique setting of a solitary right kidney due to a previous living donor left nephrectomy.

## 2. Case History

Informed consent to use case details and images was obtained from the patient.

**Figure 1:**
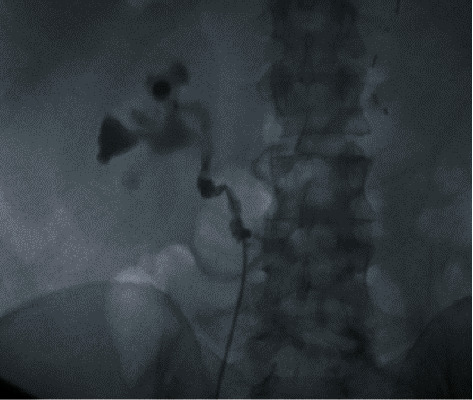
Retrograde pyelogram showed a complete occlusion (~2 centimeters in length) of the proximal ureter. Contrast examination showed a tortuous proximal ureter with kinking.

A 47-year-old Caucasian male with a past medical history of hypertension and CKD stage 3 was referred to the transplant surgery clinic by the urology service with a diagnosis of right ureteral obstruction. He had an extensive past surgical history significant for a left native kidney nephrectomy for donation 14 years prior to this presentation, partial small bowel resection due to inflammatory bowel disease, total colectomy, and right-sided inguinal hernia repair. The patient had a six-year history of CKD with baseline serum creatinine ranging 1.7-2.0 mg/dL. He had experienced episodes of acute kidney injury (AKI) which were attributed to obstructive uropathy secondary to proximal ureter stricture with no obvious etiology identified. During a more severe episode of AKI, the patient had required two sessions of hemodialysis. Seven months prior to the current presentation, his serum creatinine had increased to 12 mg/dL. A CT scan at that time showed dilation of the renal pelvis and proximal ureter. The patient had relief of his symptoms and return of his serum creatinine to baseline after a stent was placed in the right ureter at another institution. A repeat cystoscopy and retrograde pyelogram at our institution revealed a complete occlusion (~2 centimeters in length) of the proximal ureter. The catheter was advanced through the point of occlusion and contrast examination revealed the presence of a tortuous proximal ureter with kinking at the level of the ureteropelvic junction. The cause of the kinking was believed to be due to possible crossing vessels. A decision was made not to balloon dilate the stricture with a high risk of failure given the length of obstruction (Figure 1). The patient was subsequently referred to transplant surgery to discuss potential treatment options.

**Figure 2:**
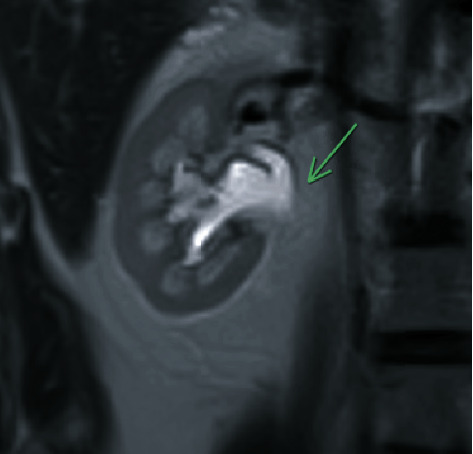
MRI shows a dilated right proximal ureter and pelvicalyceal system.

**Figure 3:**
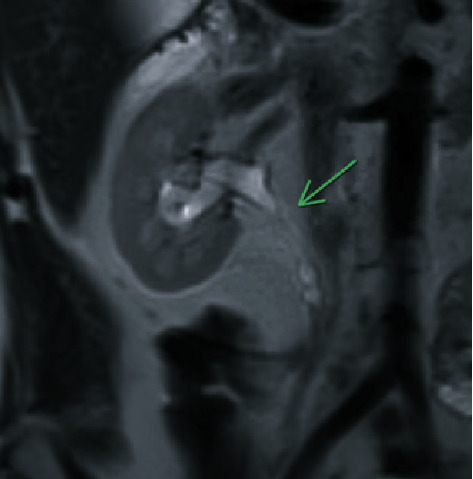
MRI shows encasement of the proximal right ureter, which intraoperatively was found to be due to a fibrous sheath.

A follow-up MRI was obtained which revealed mild pelvicaliectasis of the right kidney with ureteral narrowing in the region of the proximal ureter (Figures 2 and 3). The MRI also showed a beaded appearance of the proximal ureter. The information from the MRI was limited as it did not include the full length of the ureter in the examination. Treatment options, including chronic indwelling ureteral stent with periodic exchanges, chronic percutaneous nephrostomy tube with periodic exchanges, pyeloplasty with ileal interposition, and autologous kidney transplant, were discussed with the patient. The patient elected to proceed with a native nephrectomy and autologous kidney transplant.

The patient had a preoperative serum creatinine of 1.5 mg/dL with the indwelling ureteral stent. The patient underwent a right native nephrectomy via a 12-rib flank retroperitoneal approach. The right ureter was dissected proximal to the obstruction, the indwelling ureteral stent was removed, and the distal ureter was ligated. Of note, the ureter wall was thickened and chronically obstructed secondary to thick fibrotic tissue encasing the ureter 5 cm distal to the ureteropelvic junction. The cause of this fibrosis was believed to be the long-standing inflammatory bowel disease, rather than an indwelling ureteral stent that had not been of long enough duration. The renal vein and artery were dissected out. After systemic heparinization, the renal artery was ligated and divided. The inferior vena cava was partially clamped, the renal vein was divided at its insertion into the cava, and the kidney was removed to be flushed at the back table. The renal vein stump was closed with a running 5-0 prolene suture.

The kidney was subsequently transplanted into the right iliac fossa via Gibson incision. The renal vein and artery were anastomosed in an end-to-side fashion to the external iliac vein and artery, respectively. The vascular anastomoses were placed more distally to the iliac vessels to allow for a tension-free ureteroneocystostomy.

The bladder was then filled with methylene blue dye to help aid in identification. The anterolateral surface of the bladder was exposed, and the muscle was incised to expose the bladder mucosa. The ureter was spatulated, and an anterior ureteroneocystostomy was then constructed over the stent. The bladder muscle was then sewn over the anastomosis. The ureteric anastomosis was an extravesical nonrefluxing Litch ureteroneocystostomy. There was an estimated blood loss of 200 ml for the entire procedure. Total cold ischemia time and warm ischemia time were 3.5 hours and 1 hour, respectively. The total operative time was 8 hours 59 minutes.

The serum creatinine increased to 2.3 mg/dL on postoperative day 1; however, the serum creatine continued to trend downward over the next few days. His postoperative course was uncomplicated, and he was discharged on postoperative day 4 with a serum creatinine of 1.3 mg/dL. He remains well and at 15 months postprocedure his serum creatinine is 1.5 mg/dL with a normal duplex ultrasound of the kidney.

## 3. Discussion

KAT is an effective procedure to manage certain cases of ureteral obstruction. One of the largest reports on KAT was by Novick et al. [7]. They had a population of 108 patients who underwent KAT for multiple indications such as renal artery disease (67 patients), ureteral replacement (27 patients), and renal cell carcinoma (14 patients) [7]. They found that approximately 25 out of the 27 patients who underwent KAT for ureteral replacement did well postoperatively. Similar to our patient, all participants in that study had an immediate function of their autotransplanted kidneys and had a stable renal function on follow-up, with serum creatines ranging from 1.0 to 2.2 mg/dL [7]. In regard to long-term renal function after KAT, Ruiz et al. reported a mean serum creatinine level of 1.4 mg/dL after a mean follow-up time of 73.1 months [3]. Tran et al. reported after a median follow-up of 73.5 months 90.3% of patients had continued function of their autotransplanted kidney, including 3 patients with solitary kidneys [4].

In another multicenter study of 51 patients—5 of whom had undergone autotransplantation of a solitary kidney—the reported complication rates were on par or better than complication rates associated with other major urological operations [8]. The most common complications associated with KAT were urinary tract infection and hemorrhage [2, 3]. Higher grade complications that were associated with KAT included septic shock and venous thrombosis [8]. Cowan et al. managed graft thrombosis that occurred in the recovery area by immediately taking the patient back into the OR [8]. The exact incidence of venous thrombosis after KAT is unknown. Moghadamyeghaneh et al. in a nationwide analysis of KAT found that thrombosis of the renal vein occurred at an overall rate of 3.2%. They found no difference in the occurrence rate of venous thrombosis in patients with elective admission versus without elective admission [5]. It is important to monitor the vascular status of the transplanted kidney using imaging such as Doppler ultrasound to identify patients that may require intervention. Ruiz et al. suggest performing Doppler ultrasound the first day after surgery [3]. If there is any suspicion for thrombosis, then a CT angiography must be done. During the follow-up period in the study conducted by Ruiz et al., only 1 kidney was lost due to renal vein thrombosis [3]. This may indicate that renal vein thrombosis is not a common complication of KAT.

The review of the literature suggests that graft loss after KAT is uncommon. In a study conducted by Cowan et al., graft loss only occurred in 2 out of 54 KAT procedures [8]. Multiple studies have suggested that KAT is associated with low morbidity, but when compared to traditional allogenic kidney transplantation, it was found to have morbidity rate up to 46.2% [2, 5]. It is noteworthy that individuals who had undergone KAT for ureter pathology had the lowest morbidity rate [5].

To our knowledge, this is the first report of KAT in an individual with a solitary kidney secondary to prior kidney donation. The beauty of “giving the gift of life” with living donation brings with it the trepidation of complications or malfunction of the remaining organ. The present case is unique in that he had undergone a prior living donor nephrectomy and was deemed to have a satisfactory functioning solitary kidney, only to later develop ureteral obstruction and dysfunction of his remaining kidney. There was a necessity to perform a procedure to salvage his remaining kidney's function, but the question at hand was what that definitive procedure would be. The cause of this patient's proximal ureteral stricture remains unclear and might have been related to the patient's extensive past surgical history. Although iatrogenic injuries to the ureter are more common during gynecologic surgery, they are reported to occur at a rate of 5% to 15% after procedures involving the colon and the rectum [9, 10]. The right ureter is particularly at risk during lateral mobilization of the right colon [10]. Disease factors that can increase the incidence of iatrogenic injuries to the ureter include rectal cancer and Crohn's disease [11]. Our patient was particularly at risk due to his past medical history of inflammatory bowel disease. Crohn's disease can cause ureteral strictures, more commonly seen on the right side, secondary to chronic inflammation and fibrosis [12–14].

Renal autotransplantation, although rarely performed, has been shown to be an effective and safe therapeutic option, with stable creatinine clearance in patients with and without a functioning contralateral kidney [15].

KAT, when compared to allogenic renal transplantation, offers the benefit of forgoing immunosuppressive therapy [16]. In further comparison with allogenic deceased donor kidney transplantation, KAT avoids the warm ischemic insult inherent in donation after cardiac death and the cold ischemic time inherent in donation after brain death [17]. The cold ischemic time associated with KAT is minimal and is the time it takes to close the incision after performing the nephrectomy and repositioning the patient to a supine position for the transplantation approach.

The present case could have also undergone alternative treatments such as creation of an ileal ureter. Ileal interposition is a technique used in reconstructive urology in which a segment of the small bowel is used to replace the damaged segment of the ureter, forming a neo-ureter. Major complications of ileal interposition include urinary tract infection, anastomosis fistula, and small bowel obstruction [18]. Given the patient's extensive intraabdominal surgical history involving a previous small bowel resection, technical concerns for this approach included adhesive disease and its inherent risk of incidental enterotomy. Moreover, loss of further length of his small intestine, including the essential ileal region, could have a large negative impact on his health and quality of life, causing malnutrition and possible short bowel syndrome, which could prove to be more burdensome than his initial presentation of ureteral stricture [3]. Direct anastomosis of the renal pelvis to the bladder proved to be a sufficient option in this patient. There was no evidence of reflux nephropathy during the follow-up of our patient. Similarly, in a case study by Rajfer et al., there was no deterioration in renal function attributed to obstruction at the anastomosis site or to the free reflux between the bladder and renal pelvis when pyelovesicotomy was used as a form of urinary reconstruction in renal transplantation [19].

In conclusion, the present case and the reports in the literature suggest KAT as a good alternative procedure in cases with complex proximal ureteric lesions causing obstructive uropathy.

## References

[1] Shackman R. D. W (1963). Surgical Kidney. *British Medical Journal*.

[2] Alameddine M., Moghadamyeghaneh Z., Yusufali A. (2018). Kidney autotransplantation: between the past and the future. *Current Urology Reports*.

[3] Ruiz M., Hevia V., Fabuel J.-J., Fernández A.-A., Gómez V., Burgos F.-J. (2017). Kidney autotransplantation: long-term outcomes and complications. Experience in a tertiary hospital and literature review. *International Urology and Nephrology*.

[4] Tran G., Ramaswamy K., Chi T., Meng M., Freise C., Stoller M. L. (2015). Laparoscopic nephrectomy with autotransplantation: safety, efficacy and long-term durability. *Journal of Urology*.

[5] Moghadamyeghaneh Z., Hanna M. H., Fazlalizadeh R. (2017). A nationwide analysis of kidney autotransplantation. *The American Surgeon*.

[6] Tseng T. Y., Stoller M. L. (2009). Obstructive uropathy. *Clinics in Geriatric Medicine*.

[7] Novick A. C., Jackson C. L., Straffon R. A. (1990). The role of renal autotransplantation in complex urological reconstruction. *The Journal of Urology*.

[8] Cowan N. G., Banerji J. S., Johnston R. B. (2015). Renal autotransplantation: 27-year experience at 2 institutions. *The Journal of Urology*.

[9] Delacroix S. E., Winters J. C. (2010). Urinary tract injures: recognition and management. *Clinics in Colon and Rectal Surgery*.

[10] Ferrara M., Kann B. (2019). Urological injuries during colorectal surgery. *Clinics in Colon and Rectal Surgery*.

[11] Yellinek S., Krizzuk D. (2018). Ureteral injury during colorectal surgery: two case reports and a literature review. *Journal of the Anus, Rectum and Colon*.

[12] Sigel A., Botticher R., Wilhelm E. (1977). Urological complications in chronic inflammatory diseases of the bowel. *European Urology*.

[13] Angelberger S., Fink K. G., Schima W. (2007). Complications in Crohn's disease: right-sided ureteric stenosis and hydronephrosis. *Inflammatory Bowel Diseases*.

[14] Ueno Y., Tanaka S., Kanao H. (2006). A case of Crohn's disease with hydronephrosis caused by ureteropelvic junction obstruction. *European Journal of Gastroenterology & Hepatology*.

[15] Bourgi A., Aoun R., Ayoub E., Moukarzel M. (2018). Experience with renal autotransplantation: typical and atypical indications. *Advances in Urology*.

[16] Katabathina V., Menias C. O., Pickhardt P., Lubner M., Prasad S. R. (2016). Complications of immunosuppressive therapy in solid organ transplantation. *Radiologic Clinics of North America*.

[17] Kayler L., Yu X., Cortes C., Lubetzky M., Friedmann P. (2017). Impact of cold ischemia time in kidney transplants from donation after circulatory death donors. *Transplantation direct*.

[18] Wolff B., Chartier-Kastler E., Mozer P., Haertig A., Bitker M. O., Rouprêt M. (2011). Long-term functional outcomes after ileal ureter substitution: a single-center experience. *Urology*.

[19] Rajfer J., Koyle M. A., Ehrlich R. M., Smith R. B. (1986). Pyelovesicostomy as a form of urinary reconstruction in renal transplantation. *The Journal of Urology*.

